# Field Evaluation of the Cepheid GeneXpert *Chlamydia trachomatis* Assay for Detection of Infection in a Trachoma Endemic Community in Tanzania

**DOI:** 10.1371/journal.pntd.0002265

**Published:** 2013-07-04

**Authors:** Alexander Jenson, Laura Dize, Harran Mkocha, Beatriz Munoz, Jennifer Lee, Charlotte Gaydos, Thomas Quinn, Sheila K. West

**Affiliations:** 1 Dana Center for Preventive Ophthalmology, Wilmer Eye Institute, Johns Hopkins University, Baltimore, Maryland, United States of America; 2 International Sexually Transmitted Disease Research Laboratory, Division of Infectious Diseases, Johns Hopkins University, Baltimore, Maryland, United States of America; 3 Kongwa Trachoma Project, Kongwa, Tanzania; 4 Division of Intramural Research, National Institute for Allergy and Infectious Diseases, National Institute of Health, Bethesda, Maryland, United States of America; University of California San Diego School of Medicine, United States of America

## Abstract

**Purpose:**

To determine the sensitivity, specificity, and field utility of the Cepheid GeneXpert *Chlamydia trachomatis* (CT) Assay (GeneXpert) for ocular chlamydia infection compared to Roche Amplicor CT assay (Amplicor).

**Methods:**

In a trachoma-endemic community in Kongwa Tanzania, 144 children ages 0 to 9 were surveyed to assess clinical trachoma and had two ocular swabs taken. One swab was processed at Johns Hopkins University, Baltimore MD, using Amplicor, (Roche Molecular Diagnostics) and the other swab was processed at a field station in Kongwa using the GeneXpert *Chlamydia trachomatis*/*Neisseria gonorrhoeae* assay (Cepheid). The sensitivity and specificity of GeneXpert was compared to the Amplicor assay.

**Results:**

Of the 144 swabs taken the prevalence of follicular trachoma by clinical exam was 43.7%, and by evidence of infection according to Amplicor was 28.5%. A total of 17 specimens (11.8%) could not be processed by GeneXpert in the field due to lack of sample volume, other specimen issues or electricity failure. The sensitivity of GeneXpert when compared to Amplicor was 100% and the specificity was 95%. The GeneXpert test identified more positives in individuals with clinical trachoma than Amplicor, 55% versus 52%.

**Conclusion:**

The GeneXpert test for *C. trachomatis* performed with high sensitivity and specificity and demonstrated excellent promise as a field test for trachoma control.

## Author Summary

Trachoma, an eye infection caused by *C. trachomatis*, is the leading cause of infectious blindness worldwide, affecting the developing world. The current standard for trachoma treatment involves mass drug administration (MDA) of an antibiotic that is given to a community to reduce transmission. A field test for the presence of infection would be a useful adjunct in measuring MDA impact. However, the current standard for measuring infection involves expensive, delicate instrumentation that is often only in laboratories in developed countries or capital cities, and eye swab specimens are mostly shipped to the developed world for analysis.

This study compared a standard method for infection analysis, Roche Amplicor, in the United States, with a new test, the Cepheid GeneXpert, in the field in Tanzania. We collected 144 duplicate eye swabs in children ages 0–9 years. 12% of specimens could not be analyzed by GeneXpert due to correctable technical difficulties. Of those analyzed, 100% of samples negative by Amplicor were also negative by GeneXpert, and 95% of samples positive by GeneXpert were also positive by Amplicor. The GeneXpert was easy to use with minimal opportunities for contamination, and is a promising new test for field-testing infection in trachoma control efforts.

## Introduction

Trachoma, a chronic conjunctivitis caused by repeated infection with *Chlamydia trachomatis* (CT), is currently the leading cause of infectious blindness [1]. In recognition of the public health problem of trachoma as a Neglected Tropical Disease (NTD), the World Health Assembly passed a resolution in 1998 calling for the elimination of blinding trachoma by the year 2020 [2]. The World Health Organization (WHO) has recommended the implementation of a multi-faceted control strategy, with the acronym of SAFE, by National Trachoma or NTD Control programs. SAFE stands for surgery (to correct trichiasis), antibiotics (particularly azithromycin) to reduce the community pool of infection, face-washing programs to reduce transmission in children and environmental change to keep transmission low. With the free donation of azithromycin to endemic countries, trachoma control programs have scaled up the attempt to achieve the goal of eradication by 2020; 280 million doses of azithromycin have been provided to endemic countries since 1999 [3].

National Trachoma Control programs monitor efforts of implementing the SAFE strategy by measuring the prevalence of follicular trachoma in children ages 0 to 9 years. However, follicular trachoma can require a long time to resolve, and while there may be a rapid decline in infection following SAFE, there is often a less rapid decline in clinical disease. First reported after Mass Drug Administration (MDA) in the Azithromycin in Control of Trachoma Trial [4], infection declined at one year in Tanzania, from 20% to 7%, but the decline was less marked for clinical trachoma, which declined from 64% to 42%. This finding is not unexpected, as research in animals has reported a longer time for resolution of clinical signs following the clearance of infection [5] Several investigators working in trachoma endemic countries have reported that between 40–60% of clinical follicular trachoma seen in children may not have infection [6], [7], [8]. The proportion is even higher with successive rounds of MDA [7]. In addition, there are cases of infection that may be either sub-clinical or have not yet manifested disease in these communities [9]. Therefore, data on the prevalence of infection may be a useful adjunct to the prevalence of clinical disease in understanding the impact of programs on trachoma over time. As was seen in The Gambia, there may be instances where infection has been eliminated and only residual clinical disease is present [10].

Existing nucleic acid amplification tests, considered a gold standard for a laboratory test of infection [11], [12], [13], involves instrumentation that is expensive and requires developed laboratories not widely available in many trachoma endemic countries. Thus, there is a need for a simple, inexpensive rapid test for CT that can be performed in the field in trachoma endemic areas. The first attempt at a field test for CT was initially promising [14] but proved to not be robust under field conditions [15].

A study in the International Chlamydia Research Laboratory at Johns Hopkins compared ocular swabs tested with the Amplicor CT assay (Roche Molecular Diagnostics, Indianapolis, IN), Abbott *m*2000 RealTi*m*e CT Assay (*m*2000) (Abbott Molecular Diagnostics, Des Plains, IL) and a new test, Cepheid GeneXpert CT/NG Assay (GeneXpert) (Cepheid, Sunnyvale, CA), and found excellent concordance [16]. The GeneXpert has already been shown to be sensitive and specific in identifying genital chlamydia infections [17], and to be easy to use in a field setting. The GeneXpert platform is already in place in many developing country settings for use in diagnosing tuberculosis [18], [19]. The ease of testing specimens using the GeneXpert system without the need for expensive equipment or extensive training, along with the extremely low likelihood of contamination as experienced by the tuberculosis program, suggested that this might be an ideal field test if it could be shown to maintain high sensitivity and specificity when testing ocular chlamydia samples in the field [18], [19].

The purpose of this study was to compare the sensitivity and specificity of the GeneXpert test, as conducted in a trachoma field station in Kongwa Tanzania, against the Amplicor test carried out at the Johns Hopkins (JHU) International Chlamydia Laboratory in Baltimore, MD on specimens from the same eye of the same children. We also report on the experience of using GeneXpert in the field.

## Methods

### Ethics Statement

The study received ethical approval from the Tanzania National Institute for Medical Research and the Johns Hopkins Institutional Review Board. Written informed consent was also obtained by the parent/guardian for the inclusion of each child.

Both eyes were graded for trachoma using the WHO simplified grading scheme [20] by an experienced trachoma grader using 2.5 loupes. Trachoma was assessed as follicular trachoma (TF), the presence of at least 5 follicles size 0.5 mm on the conjunctiva and inflammatory trachoma (TI), which is the presence of severe inflammation that obscures 50% or more of the deep tarsal vessels. Ocular swabs were collected from the left upper eyelid of each index child using identical methods. A Dacron swab (Fisher HealthCare, Houston, TX) was rotated and swiped across the upper conjunctiva three times and placed dry in a vial. Vials were placed in a cooler in the field. The vial containing the swab for GenXpert testing was immediately transferred to a minus 20 degree Celsius freezer at Kongwa Trachoma Project offices at the end of the day and stored until processing. The swab for Amplicor testing was stored cold until shipped frozen within 30 days of collection to the International Chlamydia Laboratory at Johns Hopkins University (JHU). All testing occurred throughout September 2012.

### Roche Amplicor CT

The ocular specimens sent to JHU were processed for the detection of CT using the AMPLICOR CT/NG test (Roche Molecular Diagnostics, Indianapolis, IN) according to manufacturer [16] instructions. DNA extraction of the specimens was performed on the Roche MagNA Pure LC extraction robot with 200 uL of sample resulting in 100 uL of DNA elute using the MagNA Pure LC DNA Isolation Kit I (Roche Diagnostics). Extracted DNA was processed using Amplicor PCR according to manufacturer's instructions; positive and negative controls were included in all DNA extractions and PCRs. It has been previously shown that automated extraction works as well as manual extraction for CT DNA in swab specimens [21]. Samples with an optical density (OD) of over 0.8 were recorded as positive for CT, and samples with ODs of under 0.2 were recorded as negative, samples with an OD between 0.2 and 0.799 were considered equivocal. Equivocal specimens were retested in duplicate; samples that retested equivocal on two occasions were considered negative. Specimens were processed at both sites without knowledge of the results of the other site, or the trachoma status of the child.

### Cepheid GeneXpert

Dry ocular swabs were rehydrated with 1–1.2 mL of sterile molecular grade diethylpirocarbonate (DEPC) (Quality Biological Inc. Gaithersburg, MD) water prior to testing in the field. Each specimen was vortexed for 30 seconds prior to its addition to the GeneXpert CT/NG Research Use Only Assay cartridge (Cepheid Inc; Sunnyvale, CA Version 1.0 cartridge (Lot # 01905)); along with one tube (3.0 mL) of binding reagent added to the binding reagent opening of the cartridge. The cartridge was loaded onto the GeneXpert module and analyzed using the GeneXpert CT/NG Assay Version 1.0 software. The assay run time was 1 hour and 45 minutes. The GeneXpert System is a closed, self-contained, automated platform that has minimal risk of contamination. It combines on-board sample preparation with real-time PCR to deliver answers directly from unprocessed samples. Results were reported by the computer as positive or negative for chlamydia or indeterminate (Invalid, Error, or No Result). If the initial GeneXpert result was indeterminate, the specimen was re-tested one time using a new aliquot of specimen, if available, and a new GeneXpert cartridge. The assay produces an adequacy control result and an amplification control result. When either of these failed, the test was also indeterminate, and repeated. Both controls need to be amplified for a valid test result.

A cohort of 144 children aged 0–9 years, from the Chilangalizi community in Kongwa, Tanzania, were enrolled in the study during January 2012. We expected, given the trachoma rate in that community of about 50%, to have 30% positive by Amplicor Testing. We were testing a field usable potential test, and hoped to achieve at least 90% sensitivity using the Amplicor results as the ‘gold standard’. Selecting a 95% significance level and allowing for +/−10% precision, the estimated sample size was 117 children. With our effective sample size of 127 we improved the level of precision.

**Figure 1:**
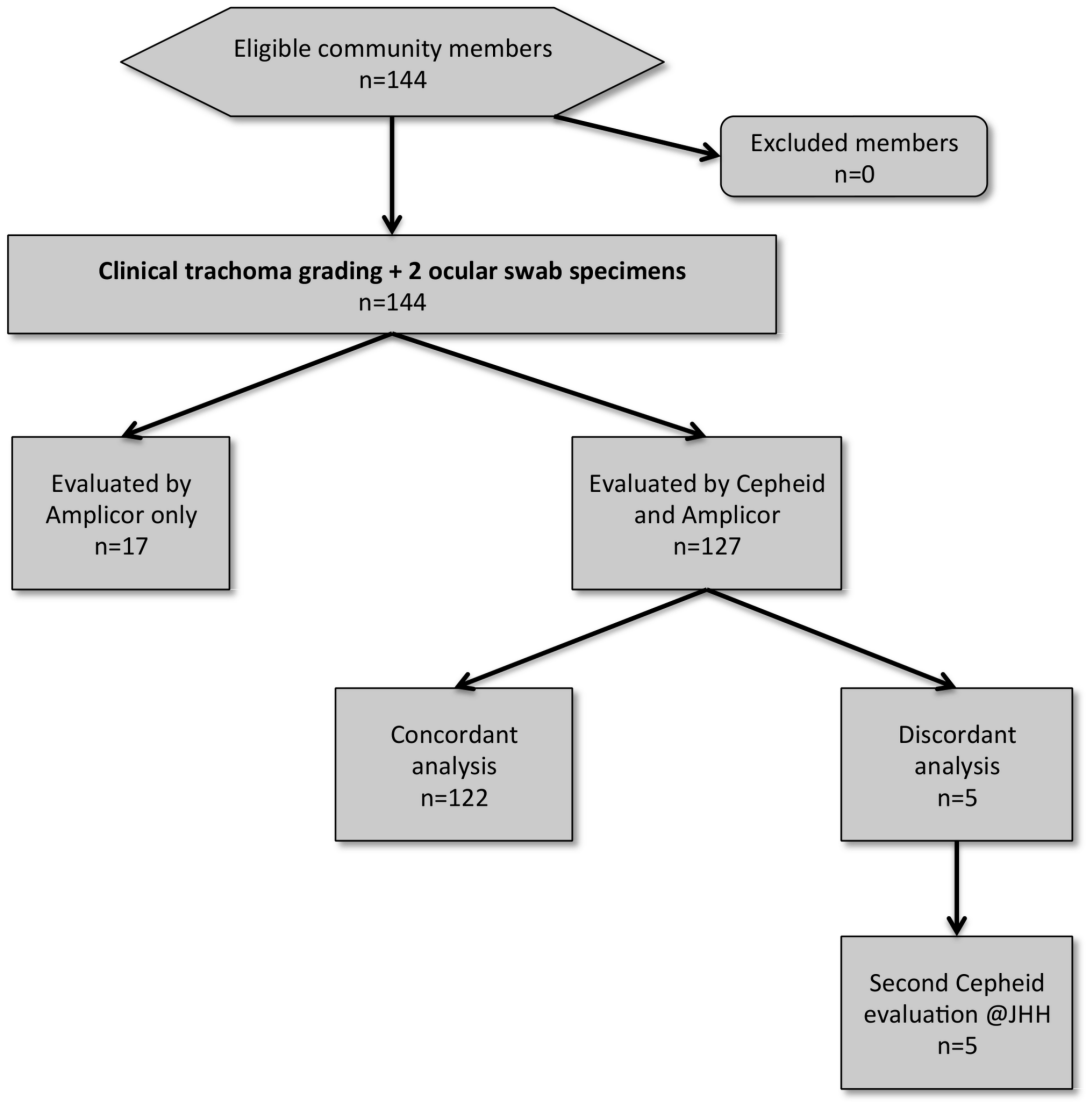
Diagram of testing for*C.* *trachomatis* using GeneXpert and Amplicor in 144 samples.****

A total of 144 paired ocular swab specimens for this study were collected from the same eye during the survey. Of those 144 eyes, a third swab from 68 eyes was collected for another study where GeneXpert testing was to also be done in the laboratory at JHU. Specimens for testing by GeneXpert were prepared and tested the same way as described in the field and at JHU (Figure 1). Data from the third swab was only used in this study to provide more information on any discordant pairs.

All results were sent to the data coordinating center at JHU. The prevalence of trachoma and ocular CT infection was determined for the 144 eye samples. The sensitivity and specificity of the GeneXpert test was compared to Amplicor and the ease of use of GeneXpert in the field was determined by examining reasons for failure to return results and anecdotal comments from the field laboratory technician (AJ). Cepheid and Amplicor test performers were blind to the corresponding test result.

## Results

At Kongwa Trachoma Project (KTP), GeneXpert testing for 144 samples returned results for 127 or 88.2% of the samples. Reasons for invalid test results included insufficient sample (***error code 5007***) in 9/144 (6.3%), other material in sample (***error code 2008***) in 4/144 (2.8%) and a sudden loss of electricity, which resulted in loss of 4/144 (2.8%) specimens. The characteristics of the children who had results from both GeneXpert and Amplicor as compared to children who had results from Amplicor only are shown in Table 1. There was no significant difference between those in the final analyses and those who did not have results from GeneXpert.

**Table 1:** Characteristics of the children in sample according to number of tests results.

Characteristic	Results from both tests N = 127	Results from Amplicor only N = 17	p-value
Age group			
% 0–3 years	52.8	35.3	0.11
% 4–5 years	33.1	58.8	
% 6 years	14.2	5.9	
% Female	48.8	58.8	0.44
Active Trachoma			
None	51.2	58.8	0.82
TF only	32.3	29.4	
TI only	3.9	5.9	
TF and TI	12.6	5.9	
AMPLICOR +	27.6	35.3	0.51
CEPHEID +	31.5	--------	

The same proportion of positive specimens was found by GeneXpert and Amplicor in children with TF, 39% and slightly more with GeneXpert in children with TI and in children without signs of trachoma (Table 2).

**Table 2:** Trachoma status and infection positive results according to the two tests.

	% PCR positive	
Active Trachoma Status	AMPLICOR +	CEPHEID +
None	(3/65) 4.6	(6/65) 9.2
TF only	(16/41) 39.0	(16/41) 39.0
TI only	(3/5) 60.0	(4/5) 80.0
TF and TI	(13/16) 81.3	(14/16) 87.5
Total	(35/127) 27.6	(40/127) 31.5

All 35 positives by Amplicor were also positive by GeneXpert, and five additional positives were found by GeneXpert that were Amplicor negative (Table 3). When compared to Amplicor the sensitivity of GeneXpert was 100% and specificity was 95%. The 5 samples that were GeneXpert positive at KTP, but negative by Amplicor were re-tested at Johns Hopkins using GeneXpert, and 2 of the 5 were positive.

**Table 3:** Correspondence between Cepheid and Amplicor.

	Amplicor	Negative	Positive	Total
Cephied				
Negative		87	0	87
Positive		5	35	40
Total		92	35	127

Sensitivity of Cepheid = 100%, specificity = 95%, positive predictive value = 88% and negative predictive value = 100%.

The field technician in Kongwa noted that GeneXpert was easy to run according to the protocol. It was also observed that due to the extreme dryness of the environment, more water was needed to ensure the volume of the specimen required for optimal performance in the Cepheid machine. Once the adjustment was made by adding 1.2 mL of DEPC water to the sample, instead of 1.0 ml, there were no more failures due to insufficient sample. It was also noted that the generator (Robin [Subaru, Japan Model RBG5000CLE, 4.5Kwatts, Freq 50 Hz) was insufficient to run the GeneXpert module during electricity failure; a generator with greater power would likely have helped avoid the 4 sample losses.

## Discussion

In 144 paired ocular samples, we evaluated the sensitivity and specificity of GeneXpert test for *C. trachomatis*, as carried out under field conditions, against the Amplicor CT PCR test, as carried out at the International Chlamydia Laboratory at Johns Hopkins University. Sensitivity and specificity were high, and the result of further testing of the GeneXpert positive/Amplicor negative specimens increased the likelihood that some of these discordant samples might have been true positives.

There is an added advantage of the GenXpert assay over other laboratory tests for CT, as the GeneXpert system includes a sample adequacy control test (SAC), which insures that there is human DNA in the sample or the test will be reported out as “indeterminate”. It also includes a specimen processing control (SPC) to indicate that amplification takes place for the SPC control, indicating that there are no PCR inhibitors present. It is theoretically possible that the negative Amplicor specimens and the positive GeneXpert specimen discordance was in part due to inadequate sampling for the Amplicor specimen. This is unlikely because all the specimens were collected the same way and none of the GeneXpert samples were indeterminate, either in the field or at JHU. Since there was no order to the samples, the chances that all the indeterminate samples were sent for Amplicor processing is low. For both tests, only one freeze/thaw cycle occurred, which could result in reduction of positive results [16].

The volume of sample used by Amplicor is only 50 µl of extracted DNA from a starting volume of 200 µl of original sample. At low prevalences, there is concern that the aliquot taken for testing may not contain chlamydia. Since the extraction and processing is all internal in GeneXpert, we do not know what volume is used for testing; only that 1.0 ml is used at the beginning. However, this was not a low prevalence community with 49% trachoma and 28% infection in the children. Thus, while a theoretical possibility, it is unlikely an explanation for the greater number of positive samples with GeneXpert.

We do not think that the loss of 17 samples during GeneXpert testing affected our results. The loss was higher among children ages 3 to 5 years, but was not statistically significant. Moreover, there was no reason to suspect bias as a result of loss of electricity, or insufficient volume of sample as these are unlikely to be related to any characteristic of a child but rather the time of the day/environment of the testing.

All that was required for field testing by GeneXpert was a freezer for the samples, a computer for the GeneXpert platform to process the assay and return results, a vortexer, DEPC water, disposable pipettes, and sterile gloves for working. At KTP a desk was set up next to the freezer for the GeneXpert equipment to provide a workspace. With GeneXpert's 4 cartridge module, about 24 samples could be processed in an eight hour work day. We estimate about 86 samples can be run in two eight hour days, but other activities can be undertaken during that time. Unlike the many steps required by laboratory personnel to run a standard PCR test, the GeneXpert requires the simple addition of water to the sample, vortexing, and removal of sample as the only steps open to contamination. The use of disposable pipettes and attention to details minimizes greatly the chance for contamination using the GeneXpert platform. For future testing a dedicated generator that could supply ample power to the platform module would provide backup power to allow for a smooth transition to generator power from state supplied electricity in the case of a power outage.

The requirement for a minimum of 1 ml of reconstituted sample limited how much left over sample could be retained without significant dilution and in our case when the test was lost due to sudden shut down in electricity or error in sample processing; it was only possible to perform a single retest of the failed specimen. In fact, we found that adding 1.2 ml eliminated errors of not enough sample, due to processing in a dry climate.

The potential cost of the test kits may dictate the use of a pooling strategy. Even at an assumed low price of $10/test, 100 tests would cost $1,000. A study to determine if pooling could be accomplished on the GeneXpert could potentially decrease costs. In addition, a university trained American researcher with computer and lab experience performed the field test in Tanzania, and while the GeneXpert is simpler than alternatives, GeneXpert remains untested in field settings by Tanzanian workers. However, the GeneXpert platform is being rolled out all over Africa as part of testing for tuberculosis, so there is no reason to suppose that positive experience would be any less so when testing for CT. Finally, the GeneXpert CT/NG assay was a research use only assay at the time of this study, but has now been approved by the Federal Drug Administration. The low cost of the processing platform, the ease of processing with readily available materials, plus our results showing the high sensitivity and specificity, suggest this approach may be ideal for a field test for trachoma control programs.

## Supporting Information

Checklist S1 STARD Checklist. Field evaluation of the Cepheid GeneXpert *Chlamydia trachomatis* assay for detection of infection in a trachoma endemic community in Tanzania.
(DOC)
